# New Systolic Array Algorithms and VLSI Architectures for 1-D MDST †

**DOI:** 10.3390/s23136220

**Published:** 2023-07-07

**Authors:** Doru Florin Chiper, Arcadie Cracan

**Affiliations:** 1 Faculty of Electronics, Telecommunications and Information Technology, “Gheorghe Asachi“ Technical University of Iasi, 700506 Iasi, Romania; chiper@etti.tuiasi.ro; 2 Technical Sciences Academy of Romania—ASTR, 030167 Bucharest, Romania; 3 Academy of Romanian Scientists—AOSR, 030167 Bucharest, Romania

**Keywords:** modified discrete sine transform, systolic arrays, pseudo-cycle convolution, pseudo-circular correlation, obfuscation technique, hardware security

## Abstract

In this paper, we present two systolic array algorithms for efficient Very-Large-Scale Integration (VLSI) implementations of the 1-D Modified Discrete Sine Transform (MDST) using the systolic array architectural paradigm. The new algorithms decompose the computation of the MDST into modular and regular computational structures called pseudo-circular correlation and pseudo-cycle convolution. The two computational structures for pseudo-circular correlation and pseudo-cycle convolution both have the same form. This feature can be exploited to significantly reduce the hardware complexity since the two computational structures can be computed on the same linear systolic array. Moreover, the second algorithm can be used to further reduce the hardware complexity by replacing the general multipliers from the first one with multipliers with a constant that have a significantly reduced complexity. The resulting VLSI architectures have all the advantages of a cycle convolution and circular correlation based systolic implementations, such as high-speed using concurrency, an efficient use of the VLSI technology due to its local and regular interconnection topology, and low I/O cost. Moreover, in both architectures, a cost-effective application of an obfuscation technique can be achieved with low overheads.

## 1. Introduction

The informational age poses multiple technical challenges for data distribution, processing, and representation, such as bandwidth and network congestion, scalability, latency and real-time communication, data synchronization and consistency, content delivery optimization, and data security and privacy [1]. High data volumes, especially for multimedia content such as videos or high-resolution images, can strain network infrastructure and result in slow or unreliable data transfer. Processing and rendering digital media content can consume significant energy, leading to reduced device runtime, while resource-constrained devices may struggle with the processing power required to efficiently encode or decode high-resolution or high-bitrate video, audio, or imaging formats [2,3].

Addressing these technical challenges often requires a combination of efficient algorithms, network protocols, infrastructure optimization, security measures, and continuous monitoring and adaptation to evolving technologies and user requirements. Key research areas such as image/video compression, decoding, accurate real-time data transmission using area-efficient, high-performance processors, and others drive innovation to meet the technical requirements for such content-rich and computationally intensive applications [4].

Certain applications require real-time or near real-time information distribution, such as telemedicine, video streaming, online gaming, or financial transactions. Minimizing latency, or the delay between sending and receiving data, is fundamental to ensuring a seamless user experience, and, thus, an effective use of VLSI technologies and innovative algorithms and architectures, implemented as either Field Programmable Gate Arrays (FPGA) or Application-Specific Integrated Circuit (ASIC), has become crucial.

Some fundamental algorithms used in audio data compression are the Modified Discrete Cosine Transform (MDCT) and the Modified Discrete Sine Transform (MDST). These transforms are using the time domain aliasing cancellation (TDAC) property [5] to avoid blocking artifacts by overlapping successive input sequences. MDCT and MDST are employed in sub-band analysis/synthesis approaches [6]. These algorithms have been used in complex filter banks in the Dolby Enhanced AC3 audio coding standard. It is important to notice that to reduce the encoding time, the MDCT and MDST spectrums should be calculated simultaneously.

Since the MDCT and MDST are computationally intensive, efficient algorithms and implementations for these transforms become important, especially on resource-constrained, battery-operated devices.

The literature describes only a few hardware implementations for the MDCT and MDST [7,8,9,10], and most of the existing algorithms on MDCT and MDST are more suitable for software implementation due to their irregular and complex computations [11,12,13,14]. However, due to the development of the VLSI technology, the cost of ASIC architectures has been reduced significantly. Moreover, such dedicated architectures can be implemented using FPGA, making these hardware implementations almost as flexible as software routines.

Our systolic array is working together with a low-cost and low-power processor where the host processor is working on input and output data management, while the hardware accelerator (the systolic array) implements the computationally intensive tasks.

In our approach, to obtain an efficient VLSI accelerator, it was necessary to restructure the basic form of the MDST algorithm in a such way that regular and modular computational structures are obtained, thus allowing for an efficient VLSI implementation using systolic arrays.

Systolic arrays have been proved to allow efficient VLSI implementations, as shown in [15,16,17,18,19]. It has been demonstrated that they best satisfy the trade-off between area and execution time for some important discrete transforms, as shown in [20].

It was already shown that the flow of the data into the algorithm is very important from a VLSI implementation point of view in a such way that communication complexity is even more important than the computational one in certain cases. Thus, regular and modular computational structures can lead to efficient VLSI implementations using distributed arithmetic [21] or systolic arrays [22]. These architectures have several merits over others, especially due to their regular and local data flow with an efficient input/output and data transfer operations, as in case of systolic arrays architectures. Thus, we have obtained efficient VLSI implementations of certain digital signal processing (DSP) algorithms that are using cyclic convolutions or circular correlations [23,24,25,26,27] that have been extended to some other regular and modular computational structures, such as, for example, skew-circular and pseudo-circular correlations or band-correlations [28,29,30].

In this paper, we have proposed two new systolic arrays for 1D MDST based on such regular and modular computational structures. One is based on pseudo-circular correlations and the other on pseudo-cycle convolutions, as both have the same form and length that allows an efficient VLSI implementation for our hardware accelerator for the computation of MDST. Moreover, since they have the same form and length, they can be computed using a single linear systolic array appropriately operated. Thus, for the first solution, the MDST can be computed in an interleaving manner, and for the second one, they are computed one after the other, leading to a reduced hardware complexity while maintaining high-speed performances specific to systolic array implementations. The obtained VLSI architecture has all the advantages of the cyclic convolution and circular correlation-based structures VLSI implementations, such as a high-speed due to using pipelining and parallelism, efficiency due to local inter-connections, and a low I/O cost. Moreover, we will show that using the proposed VLSI algorithm and architecture, we can efficiently incorporate the hardware security techniques with low overheads.

The rest of the paper is organized as follows: Section 2 presents the original systolic array algorithm for 1D MDST with a low computational complexity using regular and modular computational structures that is well adapted for an efficient VLSI implementation, as presented at the International Symposium on Electronics and Telecommunications ISETC 2022 [31], and a proposed improved version of the algorithm that allows an implementation with increased performance. Section 3 presents the proposed systolic array architecture that allows a more efficient implementation of the VLSI algorithm with a significant reduction of the hardware complexity, and which allows a more efficient incorporation of the obfuscation technique. Section 4 presents a discussion of the obtained results. In Section 5, we present the conclusions and some directions for future work.

## 2. Systolic Array Algorithms for the Computation of 1D MDST

### 2.1. A Systolic Array Algorithm for the Computation of 1D MDST [31]

The 1-D MDST is defined as:

(1) $$Y\left(k\right)=\overset{N-1}{\underset{i=0}{\sum }}x\left(i\right)\cdot \sin[\left(2i+1+N/2\right)\left(2k+1\right)\alpha /2]$$

for k=0, …, M−1, where M=N/2 and the elementary angle $\alpha =\frac{\pi }{2M}$.

As shown in [31], to reformulate the basic form of the algorithm given by the Equation (1), we have introduced some restructuring input sequences defined below.

First, we define the following auxiliary input sequences:

(2) $$x_{C}\left(i\right)=x\left(i\right)\cdot \cos[\left(2i+1+N/2\right)\alpha /2]$$

(3) $$x_{S}\left(i\right)=x\left(i\right)\cdot \sin[\left(2i+1+N/2\right)\alpha /2]$$

Using the introduced sequences, we define additional auxiliary input sequences $x_{C}^{\prime }\left(i\right)$ and $x_{C}^{\prime \prime }\left(i\right)$:

(4) $$x_{C}^{\prime }\left(i\right)={\left(-1\right)}^{i+1}\left[x_{C}\left(i\right)+x_{C}\left(N-1-i\right)\right]$$

(5) $$x_{C}^{\prime \prime }\left(i\right)=x_{C}\left(i\right)-x_{C}\left(N-1-i\right)$$

and, finally, the auxiliary input sequences $x_{a}\left(i\right)$ and $x_{b}\left(i\right)$:

(6) $$x_{a}\left(1\right)=x_{C}^{\prime }\left(0\right)$$

(7) $$x_{a}\left(i+1\right)=x_{C}^{\prime }\left(i\right)+x_{a}\left(i\right)$$

for $i=\bar{1,M-1}$.

(8) $$x_{b}\left(1\right)=x_{C}^{\prime \prime }\left(0\right)$$

(9) $$x_{b}\left(i+1\right)=x_{C}^{\prime \prime }\left(i\right)+x_{b}\left(i\right)$$

for $i=\bar{1,M-1}$.

Using these auxiliary input sequences and appropriate permutations of the indices, we can reformulate the computation of the MDST into two pseudo-cyclic convolutions, as shown in Equations (10) and (11)

(10) $$\left[\begin{array}{c} T_{a}\left(4\right)\\ -T_{a}\left(8\right)\\ -T_{a}\left(10\right)\\ T_{a}\left(6\right)\\ -T_{a}\left(12\right)\\ T_{a}\left(2\right) \end{array}\right]=\left[\begin{array}{rrrrrr} \cos32\alpha & \cos40\alpha & -\cos24\alpha & -\cos48\alpha & \cos8\alpha & -\cos16\alpha \\ -\cos40\alpha & -\cos24\alpha & \cos48\alpha & \cos8\alpha & -\cos16\alpha & \cos32\alpha \\ -\cos24\alpha & -\cos48\alpha & \cos8\alpha & \cos16\alpha & -\cos32\alpha & \cos40\alpha \\ \cos48\alpha & \cos8\alpha & -\cos16\alpha & -\cos32\alpha & \cos40\alpha & -\cos24\alpha \\ -\cos8\alpha & -\cos16\alpha & \cos32\alpha & \cos40\alpha & -\cos24\alpha & \cos48\alpha \\ \cos16\alpha & \cos32\alpha & -\cos40\alpha & -\cos24\alpha & \cos48\alpha & -\cos8\alpha \end{array}\right]\times \left[\begin{array}{c} x_{a}\left(2\right)+x_{a}\left(11\right)\\ x_{a}\left(4\right)+x_{a}\left(9\right)\\ -x_{a}\left(5\right)-x_{a}\left(8\right)\\ -x_{a}\left(3\right)-x_{a}\left(10\right)\\ x_{a}\left(6\right)+x_{a}\left(7\right)\\ -x_{a}\left(1\right)-x_{a}\left(12\right) \end{array}\right]$$

(11) $$\left[\begin{array}{c} T_{b}\left(4\right)\\ -T_{b}\left(8\right)\\ -T_{b}\left(10\right)\\ T_{b}\left(6\right)\\ -T_{b}\left(12\right)\\ T_{b}\left(2\right) \end{array}\right]=\left[\begin{array}{rrrrrr} \cos32\alpha & \cos40\alpha & -\cos24\alpha & -\cos48\alpha & \cos8\alpha & -\cos16\alpha \\ -\cos40\alpha & -\cos24\alpha & \cos48\alpha & \cos8\alpha & -\cos16\alpha & \cos32\alpha \\ -\cos24\alpha & -\cos48\alpha & \cos8\alpha & \cos16\alpha & -\cos32\alpha & \cos40\alpha \\ \cos48\alpha & \cos8\alpha & -\cos16\alpha & -\cos32\alpha & \cos40\alpha & -\cos24\alpha \\ -\cos8\alpha & -\cos16\alpha & \cos32\alpha & \cos40\alpha & -\cos24\alpha & \cos48\alpha \\ \cos16\alpha & \cos32\alpha & -\cos40\alpha & -\cos24\alpha & \cos48\alpha & -\cos8\alpha \end{array}\right]\times \left[\begin{array}{c} x_{b}\left(2\right)+x_{b}\left(11\right)\\ x_{b}\left(4\right)+x_{b}\left(9\right)\\ -x_{b}\left(5\right)-x_{b}\left(8\right)\\ -x_{b}\left(3\right)-x_{b}\left(10\right)\\ x_{b}\left(6\right)+x_{b}\left(7\right)\\ -x_{b}\left(1\right)-x_{b}\left(12\right) \end{array}\right]$$

with $T_{a}\left(k\right)$ and $T_{b}\left(k\right)$ denoting the auxiliary output sequences that can be computed using the proposed computational structures.

The matrices in Equations (10) and (11) have a particular structure, where all the elements along the secondary diagonal of the matrix or parallel to it are the same except for the sign. This structure is called a pseudo-circular correlation. This computational structure has an important advantage from a VLSI implementation point of view, as it can be efficiently implemented using the systolic array architectural paradigm. As already known, this architecture is well appropriate for an efficient VLSI implementation.

The output sequence can be recursively computed using Equations (12) and (13) as follows:

(12) $$Y\left(0\right)=\overset{N-1}{\underset{i=0}{\sum }}\left[x_{S}\left(i\right)+x_{S}\left(N-1-i\right)\right]$$

(13) Y(k)=T(k)−Y(k−1)

for k=1,…,M−1, where T(k) are additional auxiliary output sequences that can be computed as follows:

(14) $$T\left(2k\right)={\left(-1\right)}^{k}2\cdot Y_{a}\left(k\right)$$

(15) $$T\left(M-2k\right)={\left(-1\right)}^{k+1}2\cdot Y_{b}\left(k\right)$$

where the auxiliary output sequences $Y_{a}$ and $Y_{b}$ are defined below:

(16) $$Y_{a}\left(4\right)={\left(-1\right)}^{5}x_{a}\left(M\right)-T_{a}\left(4\right)\cdot \sin8\alpha \;Y_{a}\left(5\right)={\left(-1\right)}^{9}x_{a}\left(M\right)-T_{a}\left(8\right)\cdot \sin16\alpha \;Y_{a}\left(3\right)={\left(-1\right)}^{11}x_{a}\left(M\right)-T_{a}\left(10\right)\cdot \sin20\alpha \;Y_{a}\left(6\right)={\left(-1\right)}^{7}x_{a}\left(M\right)-T_{a}\left(6\right)\cdot \sin12\alpha \;Y_{a}\left(1\right)={\left(-1\right)}^{13}x_{a}\left(M\right)-T_{a}\left(12\right)\cdot \sin24\alpha \;Y_{a}\left(2\right)={\left(-1\right)}^{3}x_{a}\left(M\right)-T_{a}\left(2\right)\cdot \sin4\alpha$$

and

(17) $$Y_{b}\left(4\right)={\left(-1\right)}^{5}x_{b}\left(M\right)-T_{b}\left(4\right)\cdot \sin8\alpha \;Y_{b}\left(5\right)={\left(-1\right)}^{9}x_{b}\left(M\right)-T_{b}\left(8\right)\cdot \sin16\alpha \;Y_{b}\left(3\right)={\left(-1\right)}^{11}x_{b}\left(M\right)-T_{b}\left(10\right)\cdot \sin20\alpha \;Y_{b}\left(6\right)={\left(-1\right)}^{7}x_{a}\left(M\right)-T_{b}\left(6\right)\cdot \sin12\alpha \;Y_{b}\left(1\right)={\left(-1\right)}^{13}x_{b}\left(M\right)-T_{b}\left(12\right)\cdot \sin24\alpha \;Y_{b}\left(2\right)={\left(-1\right)}^{3}x_{b}\left(M\right)-T_{b}\left(2\right)\cdot \sin4\alpha$$

### 2.2. An Improvement of the Proposed Algorithm for the Computation of 1D MDST

To reformulate the basic form of the algorithm given by Equation (1), we have used the sequences defined in (2)–(5) and introduced modified auxiliary input sequences as compared to (6)–(9) in order to obtain the desired matrix-vector products in the following equations:

(18) $$x_{a}\left(1\right)=-x_{C}^{\prime }\left(0\right)$$

(19) $$x_{a}\left(i+1\right)={\left(-1\right)}^{i+1}x_{C}^{\prime }\left(i\right)-x_{a}\left(i\right)$$

for $i=\bar{1,M-1}$.

(20) $$x_{b}\left(1\right)=-x_{C}^{\prime \prime }\left(0\right)$$

(21) $$x_{b}\left(i+1\right)={\left(-1\right)}^{i+1}x_{C}^{\prime \prime }\left(i\right)-x_{b}\left(i\right)$$

for $i=\bar{1,M-1}$.

Using these auxiliary input sequences and appropriate permutations of the indices, we can reformulate the computation of the MDST into two pseudo-cyclic convolutions as shown in Equations (22) and (23).

(22) [Ta(4)−Ta(8)Ta(10)−Ta(6)−Ta(12)Ta(2)]=−xa(1,12)xa(2,11)xa(4,9)−xa(5,8)−xa(3,10)xa(6,7)−xa(6,7)xa(1,12)−xa(2,11)−xa(4,9)xa(5,8)xa(3,10)−xa(3,10)xa(6,7)−xa(1,12)xa(2,11)xa(4,9)−xa(5,8)xa(5,8)xa(3,10)−xa(6,7)xa(1,12)−xa(2,11)−xa(4,9)−xa(4,9)xa(5,8)xa(3,10)−xa(6,7)xa(1,12)−xa(2,11)xa(2,11)xa(4,9)−xa(5,8)−xa(3,10)xa(6,7)−xa(1,12)×[cos16αcos32αcos40αcos24αcos48αcos8α]

with $x_{a}\left(i,j\right)=x_{a}\left(i\right)-x_{a}\left(j\right)$.

(23) [Tb(4)−Tb(8)Tb(10)−Tb(6)−Tb(12)Tb(2)]=−xb(1,12)xb(2,11)xb(4,9)−xb(5,8)−xb(3,10)xb(6,7)−xb(6,7)xb(1,12)−xb(2,11)−xb(4,9)xb(5,8)xb(3,10)−xb(3,10)xb(6,7)−xb(1,12)xb(2,11)xb(4,9)−xb(5,8)xb(5,8)xb(3,10)−xb(6,7)xb(1,12)−xb(2,11)−xb(4,9)−xb(4,9)xb(5,8)xb(3,10)−xb(6,7)xb(1,12)−xb(2,11)xb(2,11)xb(4,9)−xb(5,8)−xb(3,10)xb(6,7)−xb(1,12)×[cos16αcos32αcos40αcos24αcos48αcos8α]

with $x_{b}\left(i,j\right)=x_{b}\left(i\right)-x_{b}\left(j\right)$.

The matrices in Equations (22) and (23) have been constructed such that they can be efficiently implemented using the systolic array architectural paradigm. By achieving an arrangement of the matrix elements such that the lines parallel to the main diagonal (including the main diagonal) contain elements that along the same line are equal in absolute value, one can use pseudo-cycle convolution computational structure to realize the operations in Equations (22) and (23). As previously shown [31], the pseudo-cycle convolution structure is suitable for an efficient VLSI realization.

The output sequence can be recursively computed using Equations (24) and (25) as follows:

(24) $$Y\left(0\right)=\overset{N-1}{\underset{i=0}{\sum }}\left[x_{S}\left(i\right)+x_{S}\left(N-1-i\right)\right]$$

(25) Y(k)=T(k)−Y(k−1)

for k=1,…,M−1, where T(k) can be computed as follows:

(26) $$T\left(2k\right)={\left(-1\right)}^{k}2\cdot Y_{a}\left(k\right)$$

(27) $$T\left(M-2k\right)={\left(-1\right)}^{k+1}2\cdot Y_{b}\left(k\right)$$

and $Y_{a}$ and $Y_{b}$ are defined below:

(28) $$Y_{a}\left(4\right)={\left(-1\right)}^{5}x_{a}\left(M\right)-T_{a}\left(4\right)\cdot \sin8\alpha \;Y_{a}\left(5\right)={\left(-1\right)}^{9}x_{a}\left(M\right)-T_{a}\left(8\right)\cdot \sin16\alpha \;Y_{a}\left(3\right)={\left(-1\right)}^{11}x_{a}\left(M\right)-T_{a}\left(10\right)\cdot \sin20\alpha \;Y_{a}\left(6\right)={\left(-1\right)}^{7}x_{a}\left(M\right)-T_{a}\left(6\right)\cdot \sin12\alpha \;Y_{a}\left(1\right)={\left(-1\right)}^{13}x_{a}\left(M\right)-T_{a}\left(12\right)\cdot \sin24\alpha \;Y_{a}\left(2\right)={\left(-1\right)}^{3}x_{a}\left(M\right)-T_{a}\left(2\right)\cdot \sin4\alpha$$

and

(29) $$Y_{b}\left(4\right)={\left(-1\right)}^{5}x_{b}\left(M\right)-T_{b}\left(4\right)\cdot \sin8\alpha \;Y_{b}\left(5\right)={\left(-1\right)}^{9}x_{b}\left(M\right)-T_{b}\left(8\right)\cdot \sin16\alpha \;Y_{b}\left(3\right)={\left(-1\right)}^{11}x_{b}\left(M\right)-T_{b}\left(10\right)\cdot \sin20\alpha \;Y_{b}\left(6\right)={\left(-1\right)}^{7}x_{a}\left(M\right)-T_{b}\left(6\right)\cdot \sin12\alpha \;Y_{b}\left(1\right)={\left(-1\right)}^{13}x_{b}\left(M\right)-T_{b}\left(12\right)\cdot \sin24\alpha \;Y_{b}\left(2\right)={\left(-1\right)}^{3}x_{b}\left(M\right)-T_{b}\left(2\right)\cdot \sin4\alpha$$

## 3. Systolic Array Architectures for 1D MDST

### 3.1. The VLSI Architecture for the Algorithm of Section 2.1

As shown in [31], the VLSI architecture can be obtained by mapping the Equation (10) on a linear systolic array using the design procedure proposed in [28] and the tag control mechanism [32]. The same systolic array can be obtained by mapping Equation (11). So, it is possible to use the same systolic array to compute both equations in an interleaving manner.

The proposed hardware accelerator operates alongside a low-cost and low-power host processor. The host processor is used for input and output data management, while a hardware accelerator using the systolic array can implement the computationally intensive tasks.

**Figure 1:**
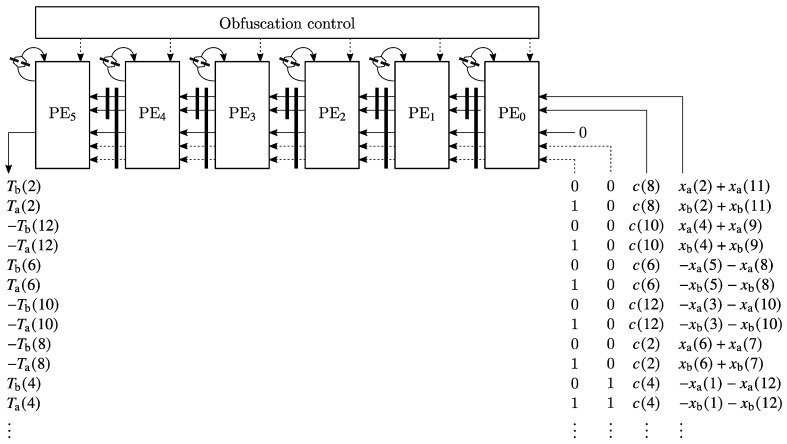
The systolic array for Equations (10) and (11) [31] © 2022 IEEE.

In Figure 1, the hardware core of the VLSI architecture that implements Equation (10) is presented. Thus, the hardware core is formed of a linear systolic array that has six elementary processors (PEs).

The post-processing stage consists of six multipliers with a constant and six adder/subtracters and implements Equations (16)–(17). The computation of the input sequences in Equations (2)–(9) and the output sequences in Equations (12)–(15) is executed on the host processor.

**Figure 2:**
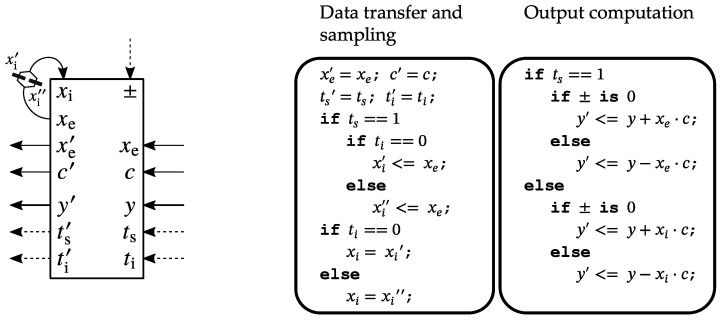
The function of a processing element from Figure 1 [31] © 2022 IEEE.

The function of the elementary processing elements (PEs) from the systolic array presented in Figure 1 is shown in Figure 2.

As explained in [31] and shown in Figure 1, the input sequence, $x_{e}$, is progressively loaded along the processing chain from right to left, starting with the processing element $PE_{0}$, and ending with the last processing element, $PE_{5}$. The $t_{s}$ sequence, also known as the tag control sequence, defines the input values sampling and storing moments within each processing element’s internal registers $x_{i}\prime$ and $x_{i}^{\prime \prime }$, which are subsequently employed in the computations. By traversing the y path of the systolic array, the partial result that is forwarded from stage-to-stage accumulates different terms of the dot products that compose the matrix-vector products of Equations (11) and (12) for the vectors $T_{a}$ and $T_{b}$, respectively. The rows of the matrix-vector products are computed in an interleaved manner, based on the state of the $t_{i}$ input.

Due to the unique characteristics of the utilized computational structure, it becomes feasible to efficiently integrate the obfuscation hardware security technique using methods similar to the ones described in [30].

As argued in [31], this solution has all the advantages of using modular and regular computational structures as cycle-convolution and circular correlation in the VLSI implementation as regularity, modularity, and local interconnections, and also a high throughput specific to systolic arrays by using pipelining and parallelism. As will be seen in the next section, it is possible to further reduce the hardware complexity without affecting the other advantages of the presented solution.

### 3.2. The VLSI Architecture for the New Algorithm of Section 2.2

**Figure 3:**
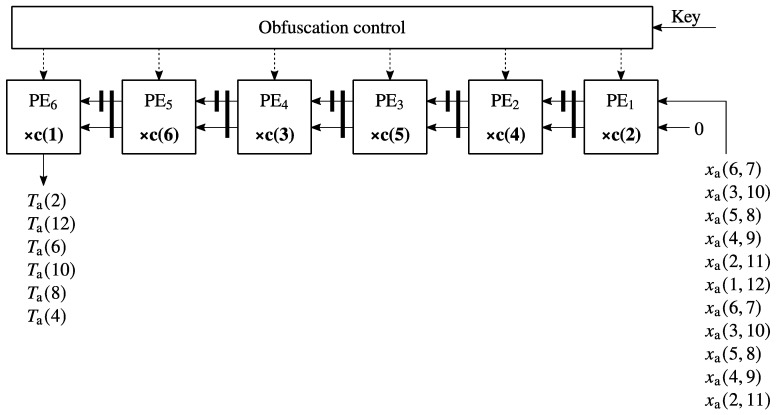
Systolic array that implements Equation (22) and also (23) but with the input sequence $x_{b}$ instead of $x_{a}$.

Using the same design method as in Section 3.1, we have obtained the systolic array from Figure 3 that can be used to compute both Equations (22) and (23). This particularity can be used to significantly reduce the hardware complexity as we can use the same linear systolic array to compute both equations. Because the same systolic array can be used to compute Equations (22) and (23) just by changing the input sequence $x_{a}\left(i,j\right)$ with $x_{b}\left(i,j\right)$, a significant reduction of the hardware complexity is achieved.

**Figure 4:**
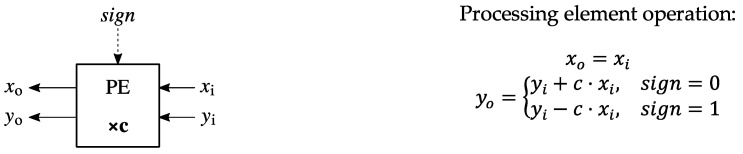
The function of a processing element (PE) used in the systolic array [33].

In Figure 4, the function of the processing elements from the systolic arrays from Figure 3 is presented. All the processing elements from Figure 3 have the same functionality, which represents an important advantage from a VLSI implementation point of view. As can be seen from Figure 4, each processing element contains a multiplier and an adder/subtracter and a MUX controlled by a tag control bit denoted as *sign* that is used to select the correct sign in the operation. One operand in each multiplier is a constant, thus allowing for a significant reduction in the hardware complexity. Compared to the processing element presented in [33], where integer constants are used for the multipliers, in this case fixed-point approximate representations of cosine coefficients are used for the low-complexity multipliers of the processing elements.

In addition to the hardware core consisting of the systolic array from Figure 3, we use a pre-processing and a post-processing stage. The pre-processing stage computes the auxiliary input sequences $x_{C}^{\prime }\left(i\right)$, $x_{C}^{\prime \prime }\left(i\right)$, using Equations (4) and (5) and $x_{a}\left(i\right)$ and $x_{b}\left(i\right)$ using Equations (18)–(21), respectively. As our systolic array is used as a hardware accelerator that works together with a host processor, Equations (2) and (3) are computed in the host processor.

The post-processing stage is used to compute the auxiliary output sequences $Y_{a}\left(k\right)$ and $Y_{b}\left(k\right)$ using Equations (28) and (29) and T(k) using Equations (26) and (27). All the multipliers in Equations (28) and (29) have one constant operand and have been implemented with additions/subtractions only. The auxiliary output sequence T(k) is sent back to the host where the output sequence Y(k) is computed using Equations (24) and (25).

We have synthesized the improved VLSI architecture from Section 3.2 using Cadence Genus 21.14 with Nangate OpenCell Library and North Carolina State University’s 15 nm FreePDK15. Table 1 summarizes the synthesis results in terms of area, power, and delay for that VLSI implementation. It can be observed that using a minimum constrained clock period the synthesis tool is able to find a solution at a clock frequency of 7.7 GHz for a delay on the critical path of 130 ps. We have a low area of 950 μm that is slowly increasing while we are increasing the clock frequency and a power of 1.25 mW at 100 MHz that is increasing linearly with the frequency. 

## 4. Discussion

The proposed two VLSI architectures presented in this paper represent the first systolic array architectures proposed until now, although using of systolic arrays in the VLSI implementations offers certain advantages, as can be seen also from this paper.

First of all, we have obtained two new systolic array algorithms for 1-D MDST that have a low hardware complexity/power consumption and allow an efficient VLSI implementation. At the same time, besides the advantage of a low hardware complexity offered by the systolic array architectural paradigm, the systolic arrays allow a high-speed performance at a reduced hardware complexity due to its low delay on the critical path. Furthermore, the proposed systolic array-based architecture enables an efficient integration of the obfuscation technique with minimal overheads. The incurred overhead due to the incorporation of the obfuscation technique consists of 6 four-way one-bit wide multiplexers, which translates in an under 1% area overhead of the total chip area. Moreover, the impact on the speed of the DCT core operation is negligible as the multiplexers are not placed on the critical data path of the systolic arrays.

For the proposed systolic arrays algorithms, we have obtained two new VLSI architecture one for each systolic array algorithm. Both systolic arrays contain only six processing elements for each one and allow the computation of the two computational structures (pseudo-circular correlation and pseudo-cycle convolutions, respectively) on a single linear systolic array, resulting a significant reduction of the hardware complexity, but the second VLSI architecture developed in Section 3.2 allows a further significant reduction of the hardware complexity and implicitly of the power consumption by replacing the general multipliers with multipliers where one operand is a constant. Due to the fact that each multiplier with a constant can be implemented using only adders and shift operations that does not imply any hardware cost besides a significant reduction in hardware complexity, the speed performances have been increased due to the fact that the delay on the critical path is only $3T_{a}$, where $T_{a}$ is the delay of one adder due to the fact that we are using only adders/subtracters and shift operation to implement our VLSI architecture. As can be seen from Table 2, to implement the constant multipliers, we need only three adders and shift operations, with only one exception where there are four such adders.

As a benefit of using the pipelining mechanism and a short critical path of only $3T_{a}$ stemming from the simple adder-only implementations of the constant multipliers, the proposed VLSI architecture offers high-speed performances, while maintaining a reduced hardware cost due to the low complexity of the multipliers. Furthermore, the described solution can accommodate with low overheads an effective integration of the obfuscation technique by using only six MUXs while maintaining the speed performances.

Additionally, both proposed solutions share the VLSI implementation benefits offered by cycle convolution and circular correlation topologies due to the regular and modular nature of these architectures, resulting in an efficient VLSI implementation while maintaining a low I/O cost.

## 5. Conclusions and Future Works

In this paper, an improvement of a previously reported systolic array algorithms for efficient VLSI implementations of the 1-D Modified Discrete Sine Transform (MDST) has been presented. Using the systolic array architectural paradigm and the proposed systolic array algorithms, low-complexity VLSI implementations of 1D MDST have been obtained. The new algorithms decompose the computation of the MDST into modular and regular computational structures called pseudo-circular correlations and pseudo-cycle convolutions that lead to efficient VLSI implementations. The second proposed algorithm can be used to further reduce the hardware complexity by replacing the general multipliers from the first one with multipliers with a constant that have a significantly reduced complexity. The resulting VLSI architecture can be used to obtain a low hardware complexity implementation with significantly higher speed performances, proving that the systolic array architectural paradigm can be used to overcome the area-speed-power tradeoff. Moreover, in both architectures, a cost-effective application of a hardware security technique can be achieved. 

One future trend that we can mention here consists of the use of the systolic array architectural paradigm to obtain VLSI implementations for some other discrete transforms with a low hardware complexity while maintaining high speed performances at the same time.

Another future trend for our work consists in using the systolic array architecture to efficiently incorporate the hardware security techniques, particularly the obfuscation technique, in other discrete transforms.

## Figures and Tables

**Table 1:** Summary of synthesis results of the VLSI core.

Constrained Clock Period/Frequency	Critical Path Delay + Setup Time [ps]	Interconnect Area [µm^2^]	Combinational Area [µm^2^]	Flop Area [µm^2^]	Total Area [µm^2^]	Equivalent Gates Count	Static Power [µW]	Dynamic Power at Constrained Clock Frequency [mW]
50 ns/20 MHz	209	238.31	358.52	311.82	935.44	46,406.71	25.8	0.0 at 20 MHz
10 ns/100 MHz	209	238.31	358.52	311.82	935.44	46,406.71	25.8	0.1 at 100 MHz
1 ns/1 GHz	213	242.55	361.51	311.82	940.60	46,662.89	26.2	1.4 at 1 GHz
300 ps/3.33 GHz	203	246.70	364.02	312.02	947.76	47,017.95	26.5	4.8 at 3.33 GHz
250 ps/4 GHz	207	246.65	363.58	312.02	947.26	46,993.09	26.5	5.7 at 4 GHz
200 ps/5 GHz	196	262.31	370.36	311.82	974.62	48,350.56	27.0	7.3 at 5 GHz
175 ps/5.71 GHz	175	279.77	380.24	311.82	1006.29	49,921.55	28.1	8.9 at 5.71 GHz
150 ps/6.67 GHz	150	308.64	405.16	312.02	1070.99	53,131.20	30.8	11.4 at 6.67 GHz
130 ps/7.69 GHz	130	346.53	461.34	312.02	1192.14	59,141.85	36.7	15.2 at 7.69 GHz

**Table 2:** Representation of the constant multiplication coefficients used in the PEs.

Coefficient (C)	Approximate Fixed-Point Value ($\hat{C}$)	$\log_{2}\left|C-\hat{C}\right|$	Representation	Number of Adders/Subtractors
cos8α	0.568359375	−11.73	$2^{-1}+2^{-4}+2^{-7}-2^{-9}$	3
cos16α	−0.355468750	−10.18	$-2^{-2}-2^{-3}+2^{-6}+2^{-8}$	3
cos24α	−0.970703125	−12.03	$-2^{0}+2^{-5}-2^{-9}$	2
cos32α	−0.748046875	−11.07	$-2^{-1}-2^{-2}+2^{-9}$	2
cos40α	0.121093750	−10.81	$2^{-3}-2^{-8}$	1
cos48α	0.884765625	−10.50	$2^{0}-2^{-3}+2^{-7}+2^{-9}$	3
sin4α	0.464843750	−13.02	$2^{-1}-2^{-5}-2^{-8}$	2
sin8α	0.822265625	−10.44	$2^{0}-2^{-2}+2^{-4}+2^{-7}+2^{-9}$	4
sin12α	0.992187500	−10.91	$2^{0}-2^{-7}$	1
sin16α	0.935546875	−10.88	$2^{0}-2^{-4}-2^{-9}$	2
sin20α	0.664062500	−10.06	$2^{-1}+2^{-3}+2^{-5}+2^{-7}$	3
sin24α	0.240234375	−10.09	$2^{-2}-2^{-7}-2^{-9}$	2

## Data Availability

Not Applicable.
